# Revisiting One-Stage Urethroplasties for Distal Urethral Strictures

**DOI:** 10.3390/jcm10245905

**Published:** 2021-12-16

**Authors:** Matthias D. Hofer, Lauren Folgosa Cooley, Ayman Elmasri, Francisco E. Martins

**Affiliations:** 1 Urology San Antonio, San Antonio, TX 78258, USA; 2 Department of Urology, Northwestern University, Chicago, IL 60611, USA; lauren.cooley@northwestern.edu (L.F.C.); ayman.elmasri@northwestern.edu (A.E.); 3 Department of Urology, School of Medicine, University of Lisbon, 1649-028 Lisbon, Portugal; faemartins@gmail.com

**Keywords:** urethral stricture, urethroplasty, urethral reconstruction, meatotomy

## Abstract

Background: Reconstructive approaches for distal urethral strictures range from simple meatotomy to utilizing grafts or flaps depending on the etiology, length and location. We describe a contemporary cohort of distal urethral strictures and report a surgical technique termed distal one-stage urethroplasty developed to address the majority of distal urethral strictures encountered. Methods: Thirty-four patients were included. The mean age was 56.7 years (range 15.7–84.9 years), the mean stricture length was 1.1 cm (0.5–1.5) and the mean follow-up was 42.5 months (28–61.3). Results: The vast majority of distal strictures (27/34 (79.4%)) were treated with our hybrid one-stage approach combining a distal urethral reconstruction with excision of the scar tissue without the need to use grafts or flaps. The average stricture length was 0.68 cm and average operative time was 24.43 min. Post-operative spraying was reported in a minority of patients (4/27 (14.8%)). The length of stricture and surgery were significantly longer in those 7/34 (20.6%) patients in whom grafts or flaps were used (2.88 cm and 154.8 min, respectively, *p* < 0.001 for both when compared to the hybrid one-stage approach). We noted 6/34 (17.6%) recurrences of distal urethral strictures, all of which were treated successfully with graft and flap repairs. Conclusions: The vast majority of distal urethral strictures are amenable to a distal one-stage urethroplasty, avoiding the use of grafts and/or flaps while achieving reasonable outcomes. This limited approach, at least initially, is associated with shorter operative time and time of catheter placement and avoids morbidity associated with graft or flap harvesting. Spraying of urine is seldomly encountered and comparable to other approaches addressing distal urethral strictures.

## 1. Introduction

Distal urethral strictures, involving the fossa navicularis and/or meatus, can be a challenging entity for reconstructive surgeons. Over the past several decades, a variety of surgical approaches to manage distal strictures have been described ranging from variations of meatoplasties to the use of grafts and flaps [1,2,3,4,5,6]. The choice of technique is dependent on the stricture etiology, anatomic location, length and the presence of fossa navicularis [1] involvement.

Strictures involving only the meatus are typically amenable to a meatotomy without requiring additional reconstructive maneuvers while still yielding a good functional and cosmetic result. For more proximal strictures with partial or complete involvement of the fossa navicularis, skin flaps and, more recently, buccal mucosa grafts, have been implemented in repairs. While buccal mucosal grafts have several advantages including the ability to be used on patients with lichen sclerosus who represent a significant percentage of distal stricture patients, skin flaps may still be necessary to avoid two-stage repairs or in complex revision surgery.

Strictures involving the meatus and distal fossa navicularis are often associated with urologic instrumentation or prolonged catheter placement [7]. Surgical repair options vary but include a variation of a meatoplasty in which the ventral urethra is incised and then the lateral edges sutured, generating a surgical hypospadias. These procedures are associated with poor cosmetic outcomes as well as disturbance in urine flow leasing to spraying. To overcome these two concerns, surgical variations to the meatoplasty technique have been reported. Malone et al. proposed removing scar tissue dorsally to avoid the hypospadias result. However, his study focused on lichen sclerosus patients who are known to scar circumferentially, which is not typically the case in post-procedural strictures were the scar is mostly ventral. [8]. Morey et al. reported on a technique of extended meatotomy in which the ventral incision is advanced proximally to include the fossa navicularis without specific reconstruction of the meatus [9]. While very effective in relieving the stricture, the cosmetic appearance can be bothersome to patients and spraying of urine is common. For strictures that extend more proximally in the urethra, Nikolavsky et al. recently proposed a transurethral approach for a buccal mucosa graft inlay which allows removal of part of the stricture while minimizing surgical meatal reconfiguration and avoiding glans transection [10,11]. While this is undoubtedly a very successful technique for distal urethral strictures longer than 1.5 cm, the majority of distal urethral strictures we encounter are shorter than this.

Given the limitations of each technique or limited indication for it, we have generated a hybrid technique, termed a distal one-stage urethroplasty, which is indicated for short (<1.5 cm) distal urethral strictures. It involves urethral scar excision and reconstruction without the use of grafts or flaps while achieving cosmetic and functional outcomes. The aim of this study was to describe a contemporary series of distal urethral strictures encountered at an academic medical center and to describe our experience with distal one-stage urethroplasties as well as our hybrid technique.

## 2. Materials and Methods

We retrospectively collected all patients diagnosed with a distal urethral stricture who underwent a urethroplasty from September 2016 to August 2019. We defined distal urethral strictures as those that involved the meatus and/or the fossa navicularis. After excluding patients with a history of hypospadias, as this is an entirely different entity given the maldevelopment of the urethra, we analyzed the surgical repair of distal urethral strictures in 34 patients.

A complete retrospective chart review of patient characteristics and their surgical procedures was performed for all patients. Recurrence was defined as a need for a revision operation due to the inability to perform a cystoscopy with a 16 French flexible cystoscope.

Statistical analysis was performed with SPSS 20 for Mac (IBM Corporation, Armonk, NY, USA) and chi-square (categorical variables) and Student’s *t*-test (continuous variables) were used. *p* < 0.05 was considered significant.

### Surgical Approach

**Figure 1:**
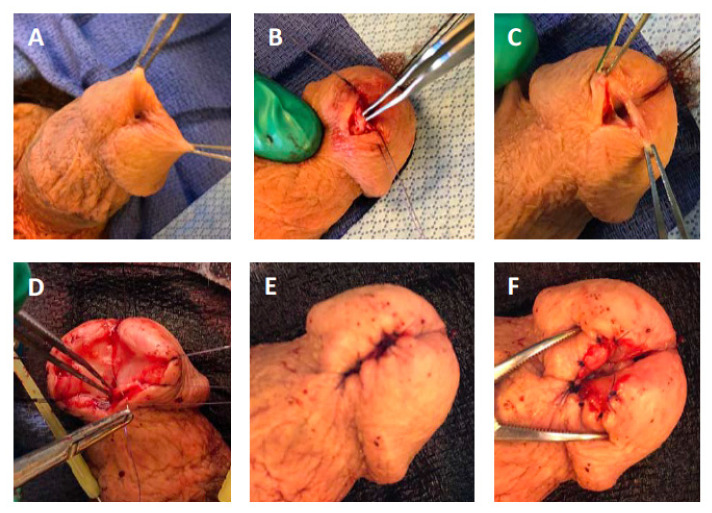
Distal one-stage urethroplasty. (**A**) Patients present with a distal stricture involving the distal fossa navicularis and meatus. The majority of patients have a history of urethral instrumentation. (**B**) The scar is located predominantly ventrally, as demonstrated. (**C**) The scar tissue is resected making an incision just underneath the urethral mucosa on glans epithelium. Removal of the scar creates a wedge-shape defect, as illustrated. (**D**) The urethral mucosa is then everted towards the glans epithelium using 5-0 PDS II sutures. (**E**) The result is a reconstructed meatus that is more slit-like than in a meatotomy. (**F**) A close up illustration of the reconstructed distal urethra.

In our hybrid approach, termed distal one-stage urethroplasty, outlined in Figure 1, the urethra is opened in its ventral midline starting at the meatus and the incision extended proximally just beyond the stricture. Next, scar tissue is removed from the ventral aspect of the urethra just underneath the mucosa while preserving the mucosa itself. Removal of the scar tissue is necessary to decrease the rate of recurrence as well as to allow reconstruction of the neomeatus by everting the urethral mucosa to the skin edge. Interrupted 5-0 PDS sutures are placed while aiming to keep their numbers to a minimum. This helps to minimize post-surgical scar formation and re-stenosis as well as the disturbance of urine flow associated with spraying. Throughout the urethral reconstruction, the distal urethra is probed with a bougie a boule to ensure patency greater than 22 French, as post-op contraction during wound healing will decrease the final size. In the seldom case of dorsal scarring, a simple incision of the dorsal urethral plate may be necessary to generate an appropriately sized meatus similar to a tubularized-incised urethroplasty [12] in which the dorsal groove will re-epithelialize without further reconstruction. It should be emphasized that while the neomeatus is moved slightly ventrally, it does not have the appearance of a surgical coronal hypospadias which minimizes post-operative spraying and helps to preserve cosmesis.

## 3. Results

### 3.1. Patient Characteristics

A total of 34 patients underwent urethroplasty for a distal urethral stricture. The mean age at surgery was 56.7 years (range 15.7–84.9 years) and the mean length of the stricture was 1.1 cm (0.5–1.5 cm). The mean follow-up was 42.5 months (28–61.3 months). Detailed patient characteristics are listed in Table 1. Among parameters associated with decreased penile perfusion, putatively conferring a risk for distal strictures and repair failure, we noted that 44.1% of our patients had a history of diabetes mellitus and 50.0% had a history of smoking (former or current), whereas the presence of peripheral vascular disease or coronary artery disease did not stand out. Regarding known risk factors for distal urethral stricture disease, a history or pathologic diagnosis of lichen sclerosus was present in 23.5% of patients, which underscores the frequency of this condition in this population.

While most of our patients (58.8%) had undergone a urologic procedure involving access to the urethra and bladder with large-sheathed instruments (transurethral resection of prostate (TURP), photovaporization of the prostate (PVP), dilations), the fraction of patients who had a Foley catheter for an extended period of time (14.7%) appeared lower than we would have expected. We also noted that the frequency of self-dilations, an unfortunate method used to maintain distal urethral patency, was low at 8.8%, while nearly a quarter of patients (23.5%) had undergone prior surgical urethral dilations.

### 3.2. Surgical Outcomes

In this study we include two cohorts with different surgical approaches; one cohort was treated with our hybrid one-stage approach, the other with grafts or flaps.

In the three years of the study, the vast majority of distal urethroplasties were one-stage approaches without the use of grafts or flaps (27/34 (79.4%)) with an average stricture length of 0.68 cm. Notably, over half of these patients (14/27 (51.8%)) underwent a prior urologic procedure with large-sheathed instruments. Post-operative spraying was reported in a minority of patients (4/27 (14.8%)). All patients were satisfied with their urination and all were able to urinate in standing position. The mean post-void residual decreased from 184.5 mL pre-operatively to 59.4 mL post-operatively. The length of surgery for the distal one-stage urethroplasty was rather short with 24.43 min (range 13–57 min). In comparison, when grafts or flaps had to be used for a more extensive repair (see below), the average time increased to 154.8 min (63–350 min) due to the more extensive dissection and graft or flap harvesting. This difference in operative length was significant (*p* < 0.001).

If the ventral urethral incision needed to be extended into the fossa navicularis, we reverted to alternative surgical approaches such as using a buccal mucosa inlay graft and/or skin flap (transverse island or Orandi flaps). Patients treated with these techniques had significantly longer mean stricture lengths (2.88 cm, *p* < 0.001). Of note, buccal inlay repairs were also necessary if we noted significant scarring in the dorsal aspect of the distal urethra and felt that solely incising the dorsal plate would be insufficient. Seven of 34 patients (20.6%) of patients were treated with only a buccal inlay graft while we utilized skin flaps in 3/34 patients (8.8%), in whom two were combined with buccal mucosa inlay grafts. 

While the distal one-stage urethroplasty was associated with a decreased length of post-operative Foley catheter (mean 7.88 days) than graft and flap repairs (21.0 days), this difference did not reach significance (*p* = 0.095).

### 3.3. Stricture Recurrences

With a mean follow-up of 26.4 months (range 12.3–45.6 months), we noted 6/34 recurrences of distal urethral strictures (17.6%). In detail, 5/27 patients (18.5%) with a distal one-stage urethroplasty had a recurrence compared to 2/7 (28.6%) treated with a buccal inlay graft and 0/3 patients who received a skin flap urethroplasty. As described above, the use of buccal mucosa inlay and skin flaps is dependent on the extent of the fibrosis proximally and dorsally; therefore a direct comparison of these reconstructive techniques appears to be not entirely valid.

Four of five patients who underwent initial one-stage urethroplasty and recurred were treated with a buccal mucosa inlay urethroplasty during the second operation. One patient received a second distal one-stage urethroplasty as he recurred within 5 weeks and the narrowing was limited to the very tip of the urethra. One recurrence that occurred in a patient with a primary buccal mucosa inlay urethroplasty was treated with a distal one-stage urethroplasty as a secondary operation. Of note, after the revision surgery, none of our patients experienced further recurrences.

We were not able to define specific risk factors for recurrence in our cohort as none of the patient characteristics we analyzed were significantly associated with stricture recurrence (all *p* > 0.05).

## 4. Discussion

Distal urethral strictures can be difficult to manage optimally in terms of both functional (e.g., urethral patency, straight urine stream) and cosmetic outcomes. Furthermore, distal stricture etiologies such as lichen sclerosus and iatrogenic trauma including dilations, which facilitate scar formation, can complicate repairs and promote stricture recurrence. Over time, a number of surgical approaches to treat these strictures have been described and include the use of buccal mucosa grafts [13,14], skin flaps [15,16] and variations of meatotomies/meatoplasties [2,3,4,5,8,9,15]. The choice of the surgical approach is mostly defined by the extent of the stricture (limited to the meatus or involving the fossa navicularis) and the etiology (e.g., skin flaps are less ideal in the presence of lichen sclerosus). In general, across all procedures, success rates of >80% have been achieved. 

Simple meatotomies and meatoplasties usually only address the meatus and, if utilized for more proximal strictures involving the fossa navicularis, as was the case in the majority of our patients, often leads to poor functional and cosmetic outcomes including urine spraying and a hypospadiac appearance. On the other hand, techniques utilizing grafts and flaps are more invasive, associated with increased patient morbidity and often more excessive than what is required to address short distal urethral strictures given our contemporary series. Therefore, our approach of a distal one stage urethroplasty without the use of grafts or flaps is an attractive option to reduce patient morbidity while still maximizing durable functional and what we feel are appropriate cosmetic outcomes. 

In the current study, we analyzed our experience of distal urethral strictures in a tertiary academic medical center over a three-year period, focusing on our surgical approach of distal-one stage urethroplasty which combines limiting surgical intervention, removing scar tissue and reconstructing the distal urethra and meatus to achieve a good cosmetic outcome and minimize the risk of spraying. Briefly, in this approach we excise the ventral scar as completely as possible between the mucosa and the glans epithelium. This maneuver is a known surgical principle in reconstructive surgery as scar removal decreases the risk of recurrence. It also allows us to evert the mucosa towards the glans epithelium given the lack of interfacing and impeding firm scar tissue, thus reconstructing a meatus which maintains a slit-like appearance, minimizing post-operative spraying and preserving cosmesis. In our cohort, 80% of strictures were limited to the meatus and distal fossa navicularis and thus were amenable to this technique. In our series, 15% of patients still experienced spraying but we believe that this number would be higher without such reconstruction of the neomeatus. In addition, spraying can be observed with any form of distal urethral reconstruction including those utilizing grafts and flaps and the rates are comparable in our observation. Another important advantage of a distal one-stage urethroplasty is the short operative time which was less than half an hour in average. This generally allows the use of monitored anesthesia care without the need of intubation and ventilation. Given that we generally consent the patient for the possibility of having to harvest flaps or grafts (in which case the patient is intubated after having started the surgery under monitored anesthesia care), we have not tried this approach under local anesthesia only. It should also be noted that we are able to limit post-operative catheter duration to 7 days (compared to 21 days with more complex procedures), which has been appreciated by our patients.

While our success rate of 82% is slightly lower than that of Morey et al., who performed an extended meatotomy and found a 88% success rate [9], we believe that the neomeatal reconstruction is cosmetically more pleasing and again, less associated with spraying, which was not reported in the respective paper. Malone et al. also specifically reported on meatal reconstruction using a combined ventral and dorsal meatal reconstruction [8], but this series only included lichen sclerosus patients which tend to have a circumferential narrowing of the meatus and distal urethra, whereas we found that the scar formation in our cohort was predominantly ventral only. Another increasingly utilized approach to reconstruct the distal urethra was introduced by Nikolavsky and colleagues [10,11], which involved the transurethral excision of a scar segment and placing a ventral buccal graft without having to open the ventral urethra. However, this approach is more suitable for strictures >1.5 cm which were less common in our contemporary patient series presenting with distal urethral strictures. Lastly, if patients recurred after our surgical approach, we achieved a 100% success rate in a second operation utilizing buccal mucosa inlays, while one patient with a nearly immediate recurrence was treated with a repeat distal one-stage urethroplasty successfully. Therefore, we feel that the advantages of limiting the initial surgical approach is warranted. It should be noted, however, that we readily implemented the use of buccal grafts as dorsal inlay and/or of skin flaps if we found that the distal stricture involved the fossa navicularis.

In our study, we also found several risk factors for the development of distal urethral strictures. Half of our patients had undergone a prior urologic procedure involving urethral access with large-sheathed urologic instruments; although this association did not reach statistical significance, it was suggested that it is a common risk factor. Additionally, half of our patients had a smoking history, while nearly half have diabetes as a comorbidity. This suggests that decreased perfusion may be associated with or contribute to the development of distal urethral strictures. On the other hand, we were not able to discern specific risk factors for recurrence, but this is likely limited by our overall sample number and the low number of recurrences.

## 5. Conclusions

In summary, we describe a contemporary series of patients presenting with distal urethral strictures. In most patients, the stricture is short (<1.5 cm) and limited to the meatus and fossa navicularis, making it amenable to a distal one-stage urethroplasty, avoiding the use of grafts and/or flaps while achieving reasonable outcomes. While not universally employable, we feel that using such a limited approach benefits patients by decreasing the morbidity associated with graft or flap harvests and longer catheterization times. Furthermore, upfront distal one-stage urethroplasty was not associated with failure if a second procedure with grafts and/or skin flaps was eventually required. 

## Figures and Tables

**Table 1:** Patient Characteristics.

Variable	Absent		Present	
	*n*	%	*n*	%
Coronary artery disease	9	26.5	25	73.5
Renal transplant	28	82.4	6	17.6
Diabetes mellitus	19	55.9	15	44.1
Peripheral vascular disease	32	94.1	2	5.9
Smoking history	17	50.0	17	50.0
History of dilations	26	76.5	8	23.5
Diagnosis of lichen sclerosis	26	76.5	8	23.5
History of urinary tract infections	29	85.3	5	14.7
History of phimosis	30	88.2	4	11.6
Prior urologic procedure	13	38.2	20	58.8
Extended Foley prior to surgery	29	85.3	5	14.7
Self-dilations	31	91.2	3	8.8
Urinary retention	31	91.2	2	5.9
Weak stream	2	5.9	2	5.9
Post void dribbling	16	47.1	17	50.0
Hypogonadism	32	94.1	2	5.9

## Data Availability

The data is available upon request from the authors.
